# Simultaneous Inversion of Particle Size Distribution, Thermal Accommodation Coefficient, and Temperature of In-Flame Soot Aggregates Using Laser-Induced Incandescence

**DOI:** 10.3390/ma17030634

**Published:** 2024-01-28

**Authors:** Junyou Zhang, Juqi Zhang, Xing Huang

**Affiliations:** 1 School of Energy and Environmental Engineering, University of Science and Technology, Beijing 100083, China; 2 Beijing Institute of Electronic System Engineering, Beijing 100854, China; zhangjq_hit@163.com; 3 Beijing Institute of Spacecraft System Engineering, Beijing 100094, China; huangxing501@163.com

**Keywords:** high temperature dispersed particles, soot aggregates, particle thermometry, inverse problem, laser-induced incandescence, LII

## Abstract

Measuring the size distribution and temperature of high-temperature dispersed particles, particularly in-flame soot, holds paramount importance across various industries. Laser-induced incandescence (LII) stands out as a potent non-contact diagnostic technology for in-flame soot, although its effectiveness is hindered by uncertainties associated with pre-determined thermal properties. To tackle this challenge, our study proposes a multi-parameter inversion strategy—simultaneous inversion of particle size distribution, thermal accommodation coefficient, and initial temperature of in-flame soot aggregates using time-resolved LII signals. Analyzing the responses of different heat transfer sub-models to temperature rise demonstrates the necessity of incorporating sublimation and thermionic emission for accurately reproducing LII signals of high-temperature dispersed particles. Consequently, we selected a particular LII model for the multi-parameter inversion strategy. Our research reveals that LII-based particle sizing is sensitive to biases in the initial temperature of particles (equivalent to the flame temperature), underscoring the need for the proposed multi-parameter inversion strategy. Numerical results obtained at two typical flame temperatures, 1100 K and 1700 K, illustrate that selecting an appropriate laser fluence enables the simultaneous inversion of particle size distribution, thermal accommodation coefficient, and initial particle temperatures of soot aggregates with high accuracy and confidence using the LII technique.

## 1. Introduction

Hydrocarbon combustion flames represent intricate dispersed mediums comprising gases and carbonaceous particles, notably soot aggregates [1]. These flames are prevalent in diverse sectors like metal smelting, petrochemicals, aerospace, and atmospheric science [2]. High-temperature soot emerges as a significant contributor to heat transfer in various industrial systems, including boilers, engines, and furnaces [3]. Beyond its role in combustion, soot serves as a crucial engineering material with applications in producing inks, dyes, and tires and promising prospects in solar cells, electronics, batteries, and quantum dots [4,5,6]. The particle size distribution and temperature evolution of soot during high-temperature processes profoundly influence both the heat transfer dynamics within industrial systems and the ultimate functional properties of the soot material [7]. Consequently, the measurement of particle size distribution and temperature of in-flame soot aggregates becomes of paramount importance.

Traditional contact measurement techniques involving intrusive probes penetrating the flame to sample soot aggregates pose risks of altering temperature and flow field distributions at the sampling site, challenging probe durability [8]. In contrast, non-contact measurement techniques such as soot spectral emission (SSE), laser-induced fluorescence (LIF), and laser-induced incandescence (LII), utilizing passive/active optical signals, emerge as uniquely suited for the extreme environmental conditions of flames [9]. 

Among these non-contact measurement techniques, LII stands out as a commonly employed method for in-situ characterization of volume fraction and particle size distribution of in-flame soot aggregates [10]. LII-based particle sizing involves exposing soot particles to nanosecond pulsed lasers, elevating them to incandescent temperatures (~3000 K), and generating LII signals [9,10]. Over the past two decades, LII technology has witnessed notable advancements [11,12]. Initially designed for pointwise (0D) or line-wise (1D) measurements, it has evolved to facilitate 2D imaging of planar LII (PLII) signals and even 3D volumetric LII (VLII) signal reconstruction [13,14,15]. Spectral resolution has transcended the traditional two-wavelength approach, with LII signals now measured at three or more wavelengths, imparting richer information and reducing susceptibility to artifacts [16,17]. In terms of temporal resolution, time-resolved laser-induced incandescence (TiRe-LII) technology provides nanosecond temporal precision, enabling the deduction of particle size distribution based on the cooling rates of soot particles [18,19]. Significant innovations, such as single-shot laser-sheet compressed ultrafast photography (LS-CUP), have further pushed the boundaries of temporal resolution in planar LII imaging to the sub-nanosecond level [20]. Collectively, these advancements in LII technology deliver informative signals with spatial resolutions spanning from 0D to 3D, wavelengths covering two to the full spectra, and temporal resolutions extending from nanoseconds to sub-nanoseconds. The recent advancements in measurement techniques have augmented the information content of TiRe-LII signals, facilitating the extraction of more details about particles and increasing the potential for simultaneously inverting multiple parameters.

In terms of multi-parameter inversion using TiRe-LII signals, the essence of LII-based particle sizing hinges on interpreting the TiRe-LII signal through heat transfer modeling [10]. Assuming a log-normal distribution of soot particle sizes, LII-based particle sizing formulates a binary inverse problem—estimating the mean (*μ*_d_) and standard deviation (*σ*_d_) of the log-normal distribution [21,22]. The ill-posed nature of the inverse problem amplifies bias in predetermined model parameters, leading to a decline in the accuracy and confidence of the inversion results for particle size distribution [23,24]. Among these predetermined model parameters, the uncertainty in the thermal accommodation coefficient (TAC) has garnered attention due to its correlation with factors such as ambient temperature, fuel type, and combustion conditions [10]. Moreover, for modeling the LII signal of in-flame soot, flame temperature (equivalent to the initial temperature of the soot before laser heating) is a requisite model parameter [25,26]. Given that LII is not commonly employed for flame temperature measurements, supplementary techniques are required to obtain both the soot particle size distribution and flame temperature simultaneously. For instance, combining two-color pyrometry (2CP) with LII enables simultaneous optical diagnosis of flame temperature and soot particle size distribution [10]. However, in the absence of supplementary techniques or reliable prior knowledge of flame temperature, the application of LII technology in flames faces challenges. 

For this scenario, this study aims to explore an alternative solution, namely, the simultaneous inversion of flame temperature, particle size distribution, and thermal accommodation coefficients using only TiRe-LII information. This approach not only eliminates the need for additional equipment but also achieves synchronized measurement of soot particle size distribution and flame temperature. In previous studies, Lehre et al. [27,28] simultaneously inferred flame temperature and the mean particle size of soot from TiRe-LII signals. However, the remaining model parameters, including the standard deviation of the particle size distribution and the thermal accommodation coefficient, were predetermined, essentially solving a binary inverse problem. In contrast, our study allows for the additional inversion of the standard deviation of particle size distribution and thermal accommodation coefficient from TiRe-LII signals without prior knowledge of flame temperature and the thermal accommodation coefficient, which is essentially a four-variable inverse problem. Given that, the purpose of this study is to introduce a four-parameter inversion strategy for in-flame soot, relying solely on TiRe-LII signals. This approach seeks to simultaneously determine the soot particle size distribution, thermal accommodation coefficient (a key thermal property of soot), and flame temperature (equivalent to the initial temperature of soot before laser irradiation). This approach does not require the independent determination of the standard deviation of particle size distribution and the thermal accommodation coefficient using other measurement techniques, thus broadening the scenarios in which LII technology can be applied independently. The LII-based four-parameter inversion strategy proposed in this study holds significance for soot research under high-temperature conditions. It presents potential applications in various scenarios, including lab-scale flames, internal combustion engines, exhaust emissions, the ambient atmosphere, and nanoparticle production. This study systematically investigates and evaluates this multi-parameter inversion strategy from various perspectives, including (1) response properties of heat transfer sub-models to flame temperature rise, (2) perturbation of flame temperature bias on LII-based particle sizing, and (3) the performance of the multi-parameter inversion strategy under different flame temperature conditions.

The paper is organized as follows: Section 2 briefly describes the LII model that drives the LII-based multi-parameter inversion strategy, in particular heat transfer sub-models. Section 3 outlines the operation details of the multi-parameter inversion strategy. Section 4 numerically analyzes the effect of flame temperature rise on the heat transfer sub-models, reveals the sensitivity of LII-based particle sizing to flame temperature deviations, and presents numerical results of the multi-parameter inversion for two typical flame temperatures. Section 5 summarizes the main conclusions.

## 2. TiRe-LII Model

**Figure 1:**
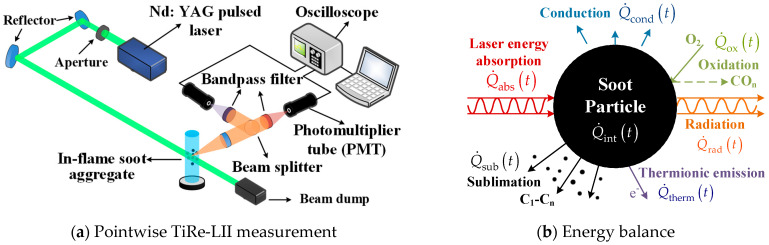

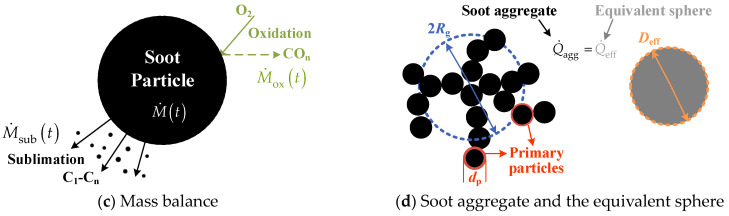
Illustration of (**a**) pointwise TiRe-LII measurement [10], (**b**) energy balance, and (**c**) mass balance in a laser-energized soot particle. Additionally, (**d**) a soot aggregate and its corresponding equivalent sphere with the same heat transfer rate.

Figure 1a depicts a classical configuration for pointwise time-resolved laser-induced incandescence (TiRe-LII) measurements. The pointwise measurement of the LII signal of laser-heated soot particles is generally achieved by the fast photomultiplier tubes (PMTs, typically <1 ns response time) combined with ultra-high-frequency oscilloscope (such as 1 GHz sampling frequency) to allow for the nanoscale temporal resolution of the TiRe-LII signal. Furthermore, the TiRe-LII signals are measured spectrally at specific wavelengths using appropriate filters. The TiRe-LII signals emitted by laser-energized soot aggregates are typically simulated by the model that integrates several heat transfer sub-models with a spectroscopic sub-model. A concise overview of the TiRe-LII model is presented here; for more comprehensive details, please refer to our previous work [29,30].

### 2.1. Heat Transfer Sub-Models

#### 2.1.1. Energy and Mass Balance Equations

As depicted in Figure 1b,c, the interaction between laser and soot aggregates involves various heat transfer processes. While the figure illustrates spherical particles as an example, the involved heat transfer processes apply similarly to laser-irradiated soot aggregates. Consequently, the instantaneous temperature *T*_p_(*t*) and diameter *d*_p_(*t*) of soot aggregates are derived by solving the energy balance equation (Figure 1b) and mass balance equation (Figure 1c) [10]:

(1) $$\dot{U}\left(t\right)={\dot{Q}}_{\mathrm{abs}}\left(t\right)+{\dot{Q}}_{\mathrm{cond}}\left(t\right)+{\dot{Q}}_{\mathrm{rad}}\left(t\right)+{\dot{Q}}_{\mathrm{sub}}\left(t\right)+{\dot{Q}}_{\mathrm{therm}}\left(t\right)+{\dot{Q}}_{\mathrm{ox}}\left(t\right)$$

(2) $$\dot{M}\left(t\right)={\dot{M}}_{\mathrm{sub}}\left(t\right)+{\dot{M}}_{\mathrm{ox}}\left(t\right)$$

where $\dot{U}\left(t\right)$ is the temporal change of internal energy of a soot particle at time *t*, with the unit of J/s; ${\dot{Q}}_{\mathrm{abs}}\left(t\right)$ , ${\dot{Q}}_{\mathrm{cond}}\left(t\right)$ , ${\dot{Q}}_{\mathrm{rad}}\left(t\right)$ , ${\dot{Q}}_{\mathrm{sub}}\left(t\right)$ , ${\dot{Q}}_{\mathrm{therm}}\left(t\right)$ , and ${\dot{Q}}_{\mathrm{ox}}\left(t\right)$ represent the heat transfer rate of soot particle caused by laser energy absorption, conduction, radiation, sublimation, thermionic emission, and oxidation at time *t*, respectively, with the unit of J/s; $\dot{M}\left(t\right)$ is the temporal of soot particle mass; ${\dot{M}}_{\mathrm{sub}}\left(t\right)$ , and ${\dot{M}}_{\mathrm{ox}}\left(t\right)$ denote the temporal change of soot particle mass caused by sublimation, and oxidation at time *t*, respectively, with the unit of g/s. In contrast to the comprehensive TiRe-LII model proposed by Michelson [10,31], this study neglects the impact of soot annealing during and after laser pulses in Equation (1) due to its unclear mechanism [32].

The soot studied in this paper is irregularly shaped aggregate particles. Taking an aggregate illustrated in Figure 1d as an example, its morphology can be described by $N_{p}=k_{f}{\left(2R_{g}/d_{p}\right)}^{D_{f}}$ [33]. In this equation, *N*_p_ is the number of sphere-like primary particles constituting the aggregate; *k*_f_ is the fractal prefactor; *R*_g_ is the gyration radius; *D*_f_ is the fractal dimension. The *D*_f_ value, ranging from 1 to 3, reflects the efficiency of occupying space by the aggregate. A higher *D*_f_ value indicates a more compact aggregate structure.

#### 2.1.2. Internal Energy of Soot Aggregates

For a soot aggregate consisting of *N*_p_ primary particles, the internal energy term is expressed as [34]:

(3) $$\dot{U}\left(t\right)=N_{p}c_{s}\rho_{s}\frac{\pi d_{p}^{3}\left(t\right)}{6}\frac{dT_{p}\left(t\right)}{dt}$$

where *c*_s_ is the specific heat of soot in J/(g·K), and the temperature-dependent fitting polynomial shown in Equation (4) was used in this study; *ρ*_s_ = 2.3031–7.3106 × 10^−5^ × *T*_p_ is the density of soot, with the unit of g/cm^3^ [34].

(4) $$\begin{array}{c} c_{s}=\left(\frac{R}{12.01\;g/\mathrm{mol}}\right)\{1.115\times {\left(\frac{597\;K}{T_{p}\left(t\right)}\right)}^{2}\exp\left(\frac{597\;K}{T_{p}\left(t\right)}\right){\left[\exp\left(\frac{597\;K}{T_{p}\left(t\right)}\right)-1\right]}^{-2}\\ +1.789\times {\left(\frac{1739\;K}{T_{p}\left(t\right)}\right)}^{2}\exp\left(\frac{1739\;K}{T_{p}\left(t\right)}\right){\left[\exp\left(\frac{1739\;K}{T_{p}\left(t\right)}\right)-1\right]}^{-2}+\frac{T_{p}\left(t\right)}{8620\;K}\} \end{array}$$

where *R* = 8.3145 J/(mol·K) is the universal gas constant.

#### 2.1.3. Heat Conduction Sub-Model

The mean free path of surrounding gas *λ*_MFP_ is expressed as [31]:

(5) $$\lambda_{\mathrm{MFP}}=\frac{k_{p}T_{g}}{\sqrt{2}\sigma_{a}P_{a}}$$

where *k*_p_ = 1.3626 × 10^−22^ atm·cm^3^/K is the Boltzmann constant in effective pressure units; *T*_g_ is the temperature of surrounding air; *σ*_a_ = 4.21 × 10^−15^ cm^2^ is the mean molecular cross section of surrounding air; *P*_a_ is the pressure of surrounding air.

For in-flame soot, the mean free path *λ*_MFP_ is equal to 389 nm at a flame temperature of *T*_g_ = 1700 K and atmospheric pressure of *P*_a_ = 1 atm, which significantly exceeds the diameter of the soot primary particles. Therefore, the heat conduction sub-model is in a free-molecular-flow regime [10,34,35]:

(6) $${\dot{Q}}_{\mathrm{cond}}\left(t\right)=-\pi D_{\mathrm{eff}}^{2}\left(t\right)\alpha_{T}\frac{P_{a}}{8}\sqrt{\frac{8R_{m}T_{g}}{\pi W_{a}}}\left(\frac{\gamma +1}{\gamma -1}\right)\left[\frac{T_{p}\left(t\right)}{T_{g}}-1\right]$$

where *D*_eff_ is the diameter of an equivalent sphere with the same heat transfer surface area as the aggregate (Figure 1d), with unit of nm (nanometer); *D*_eff_ is related to the primary soot particle diameter *d*_p_ and the aggregate size *N*_p_ through $D_{\mathrm{eff}}=d_{p}{\left(N_{p}/k_{h}\right)}^{1/D_{h}}$ , with *k*_h_ = 1.2 and *D*_h_ = 2.2 [35] for a fractal aggregate with *k*_f_ = 2.3 and *D*_f_ = 1.78 [36]; *α*_T_ = 0.37 is the thermal accommodation coefficient (TAC), which is a dimensionless parameter of particular interest in this study due to its inherent uncertainty in prior values; *P*_a_ = 1 atm is the pressure of ambient air; *R*_m_ = 83.145 g·m^2^/(mol·K·s^2^) is the universal gas constant in effective mass units; *γ* = 1.3 is the dimensionless specific heat ratio under flame temperature condition; *T*_p_(*t*) is the temperature of laser-heat soot at time *t*, with the unit of K (Kelvin); *T*_g_ = 1700 K is the temperature of ambient combustion gas, namely the flame temperature, also with the unit of K; notably, *T*_g_ is also equivalent to the initial temperature (*T*_p, 0_) of soot aggregates before laser heating; *W*_a_ = 28.74 g/mol is the molecular weight of air. It is worth noting that, in the case of atmospheric pressure flames considered in this study, the Fuchs model based on the boundary sphere is also applicable [10]. In order to enhance the computational efficiency of solving the inverse problem, a more straightforward free molecular flow model was adopted in this study.

#### 2.1.4. Sublimation Sub-Model

The temporal change rate of energy and mass of soot aggregates due to sublimation can be expressed as [34]:

(7) $${\dot{Q}}_{\mathrm{sub}}\left(t\right)=-\frac{\Delta H_{v}}{W_{v}}{\dot{M}}_{\mathrm{sub}}\left(t\right)$$

(8) $${\dot{M}}_{\mathrm{sub}}\left(t\right)=N_{p}\frac{-\pi d_{p}^{2}\left(t\right)W_{v}\alpha_{M}p_{v}}{R_{p}T_{p}\left(t\right)}\sqrt{\frac{R_{m}T_{p}\left(t\right)}{2\pi W_{v}}}$$

where *N*_p_ is the number of sphere-like primary particles that constitute a soot aggregate; *α*_M_ = 0.77 is the mass accommodation coefficient of sublimated carbon cluster; *R*_p_ = 83.145 bar·cm^3^/(mol·K) is the universal gas constant in the effective pressure units; Δ*H*_v_, *W*_v_, and *p*_v_ is the average enthalpy of formation, average molecular weight, and saturation partial pressure of subliming carbon clusters, respectively.

The temperature-dependent expressions of Δ*H*_v_, *W*_v_, and *p*_v_ are as follows [34]:

(9) $$\Delta H_{v}=2.054\times 10^{5}+7.366\times 10^{2}T_{p}\left(t\right)-0.407T_{p}^{2}\left(t\right)+1.199\times 10^{-4}T_{p}^{3}\left(t\right)-1.795\times 10^{-8}T_{p}^{4}\left(t\right)+1.072\times 10^{-12}T_{p}^{5}\left(t\right)$$

(10) $$W_{v}=17.179+6.865\times 10^{-4}T_{p}\left(t\right)+2.996\times 10^{-6}T_{p}^{2}\left(t\right)-8.595\times 10^{-10}T_{p}^{3}\left(t\right)+1.049\times 10^{-13}T_{p}^{4}\left(t\right)$$

(11) $$p_{v}=\exp\left[-122.96+9.056\times 10^{-2}T_{p}\left(t\right)-2.764\times 10^{-5}T_{p}^{2}\left(t\right)+4.175\times 10^{-9}T_{p}^{3}\left(t\right)-2.488\times 10^{-9}T_{p}^{4}\left(t\right)\right]$$

#### 2.1.5. Thermionic Emission Sub-Model

The energy change rate of soot particles caused by thermionic emission is expressed as follows [34]:

(12) $${\dot{Q}}_{\mathrm{therm}}\left(t\right)=N_{p}\frac{4\Phi m_{e}{\left[\pi d_{p}\left(t\right)k_{B}T_{p}\left(t\right)\right]}^{2}}{h^{3}}\exp\left[\frac{-\Phi }{k_{B}T_{p}\left(t\right)}\right]$$

where Φ = 7.37 × 10^−19^ J is the work function; *m*_e_ = 9.1095 × 10^−35^ J·s^2^/cm^2^ is the electron mass; *h* = 6.626 × 10^−34^ J·s is Planck’s constant; *k*_B_ = 1.381 × 10^−23^ J/K is the Boltzmann constant.

### 2.2. Spectroscopic Sub-Model

For optically thin, polydisperse soot aggregates aerosols, the spectral TiRe-LII emitted by laser-heated soot at an instant *t*, denoted as *J_λ_*(*t*), is calculated as follows [37,38]:

(13) $$J_{\lambda }\left(t\right)=\Lambda \int p\left(d_{p}\right)C_{\mathrm{abs},\;\lambda }\left[d_{p}\left(t\right)\right]I_{b,\;\lambda }\left[T_{p}\left(t\right)\right]d\left(d_{p}\right)$$

where Λ is the intensity scaling factor, accounting for the effect of soot volume fraction, geometry, and collection efficiency of detectors; *p*(*d*_p_) is the probability density function of the polydisperse primary particles of soot aggregates; *C*_abs,_ *_λ_* is the absorption cross-section of a primary particle at wavelength *λ*, which depends on the *d*_p_; *I*_b,_ *_λ_* is the blackbody spectral intensity emitted by the soot aggregates at temperature *T*_p_.

This study assumes that *d*_p_ is constant within aggregates but follows a narrow log-normal distribution between aggregates [39]:

(14) $$p\left(d_{p}\right)=\frac{1}{d_{p}\sqrt{2\pi }\ln\sigma_{d\;}}\exp\left[-{\left(\frac{\ln d_{p}-\ln\mu_{d}}{\sqrt{2}\ln\sigma_{d}}\right)}^{2}\right]$$

where *μ*_d_ and *σ*_d_ are the mean value and standard deviation of *p*(*d*_p_), respectively.

The absorption cross-section of soot aggregates is given by [33]:

(15) $$C_{\mathrm{abs},\;\lambda }\left[d_{p}\left(t\right)\right]=\frac{\pi^{2}d_{p}^{3}E\left(m_{\lambda }\right)}{\lambda }$$

where *m_λ_* is the complex refractive index (optical constant) of soot at wavelength *λ*; E(mλ)=Im|(mλ2−1)/(mλ2+2)| is the spectral absorption function of *m_λ_*, and the symbol Im|·| indicates the extraction of imaginary parts [34].

The blackbody spectral intensity *I*_b,_ *_λ_* follows the Planck law, and its integral over all solid angles is expressed as [33]:

(16) $$I_{b,\;\lambda }\left(T_{p}\right)=\frac{8\pi hc^{2}}{\lambda^{5}\cdot \left[\exp\left(\frac{hc}{\lambda k_{B}T_{p}}\right)-1\right]}$$

where the *c* = 2.998 × 10^8^ m/s is the speed of light.

## 3. Inverse Problem

### 3.1. Equivalent Thermal Accommodation Coefficient

Based on the TiRe-LII model, inferring the parameters of interest (in this study, two particle size distribution parameters, flame temperature, and thermal accommodation coefficient, totaling four parameters) from the measured TiRe-LII signals essentially involves solving an inverse problem. In order to realize the multi-parameter inversion strategy proposed in this study, a special treatment of the thermal accommodation coefficients is required. For soot aggregates, the implementation of the heat conduction mechanism (Equation (6)) depends on the determination of the equivalent heat transfer cross-section $\pi D_{\mathrm{eff}}^{2}$ . Due to the aggregate structure, heat transfer between aggregated primary particles is hindered, resulting in a shielding effect. This shielding effect is modulated by a shielding factor *η* ranging between 0 and 1, then $\pi D_{\mathrm{eff}}^{2}\alpha_{T}=\alpha_{T}\cdot \eta \pi N_{p}d_{p}^{2}$ . Importantly, the exact value of the shielding factor depends on the morphology of the aggregate. The morphology of soot aggregates in a flame is influenced by a number of factors, including fuel type, flame temperature, and combustion time, which introduces significant uncertainty into the shielding factor. Measuring the aggregate morphology in advance to determine *D*_eff_ is critical for inverting the flame temperature and particle size distribution from the LII signal when the value of the shielding factor is unknown. However, even with the support of other measurement techniques, achieving accurate *D*_eff_ remains extremely challenging. To address these challenges, we introduce a combined parameter, *α*_eff_, which combines the thermal accommodation coefficient (*α*_T_) and the shielding factor (*η*), then $\left(\eta \cdot \alpha_{T}\right)\pi N_{p}d_{p}^{2}=\alpha_{\mathrm{eff}}\pi N_{p}d_{p}^{2}$ . By inverting this combined parameter along with flame temperature and particle size distribution from LII signals, the aforementioned challenges are avoided.

In the proposed multi-parameter inversion strategy, we derive an equivalent thermal accommodation coefficient, *α*_eff_, from the normalized form of TiRe-LII signals (for a detailed derivation, refer to our previous work, Ref. [29]). By employing *α*_eff_, the product $\pi D_{\mathrm{eff}}^{2}\alpha_{T}$ in Equation (6) is replaced with $\pi N_{p}d_{p}^{2}\alpha_{\mathrm{eff}}$ :

(17) $${\dot{Q}}_{\mathrm{cond}}\left(t\right)=-\pi N_{p}d_{p}^{2}\left(t\right)\alpha_{\mathrm{eff}}\frac{P_{a}}{8}\sqrt{\frac{8R_{m}T_{g}}{\pi W_{a}}}\left(\frac{\gamma +1}{\gamma -1}\right)\left[\frac{T_{p}\left(t\right)}{T_{g}}-1\right]$$

where *N*_p_ typically follows a log-normal distribution among aggregates:

(18) $$p\left(N_{p}\right)=\frac{1}{N_{p}\sqrt{2\pi }\ln\sigma_{N\;}}\exp\left[-{\left(\frac{\ln N_{p}-\ln\mu_{N}}{\sqrt{2}\ln\sigma_{N\;}}\right)}^{2}\right]$$

where *μ*_N_ and *σ*_N_ are the mean value and standard deviation of the probability density function *p*(*N*_p_), respectively.

The normalized TiRe-LII signals are simplified using the *α*_eff_ (see Appendix A for the full derivation) as follows:

(19) $$b\left(t,\;\lambda \right)=\frac{\int N_{p}J_{\lambda }\left(t\right)dN_{p}}{\int N_{p}J_{\lambda }\left(t_{m}\right)dN_{p}}=\int \frac{d_{p}^{3}\left(t\right)p(d_{p})}{\exp\left[hc/\lambda k_{B}T_{p}\left(t\right)\right]-1}dd_{p}/\int \frac{d_{p}^{3}\left(t_{m}\right)p(d_{p})}{\exp\left[hc/\lambda k_{B}T_{p}\left(t_{m}\right)\right]-1}dd_{p}$$

where *t*_m_ is the time at which the spectral TiRe-LII signal *J_λ_*(*t*) reaches its maximum value, **b**(*t*, *λ*) is the spectral normalized TiRe-LII signal at time *t*, and the collection of **b**(*t*, *λ*) at multiple times is expressed in vector form as **b**.

Therefore, the introduction of the equivalent TAC, *α*_eff_, transforms the multi-parameter inversion based on the normalized LII signal, eliminating the reliance on prior information about the specific aggregate structure and replacing it with an estimation of the unknown variable *α*_eff_.

### 3.2. Multi-Parameter Inversion Strategy

Upon examining the heat transfer sub-models presented in Section 2, it becomes apparent that the flame temperature *T*_g_, i.e., the initial temperature *T*_p, 0_ of the soot aggregates before laser heating, is a crucial model parameter. Previous studies have highlighted the sensitivity of modeling TiRe-LII signals to deviations in flame temperature [25,26]. Consequently, precise measurement of flame temperature is paramount to ensuring the accuracy of LII-based particle sizing. While obtaining flame temperature information through alternative measurement methods introduces additional uncertainties and requires more complex devices, this paper proposes a novel multi-parameter inversion strategy. This strategy simultaneously inverts the flame temperature along with the particle size distribution parameters and the equivalent thermal accommodation coefficient from the TiRe-LII signals. Thus, the variable vector **x** of the multi-parameter inversion comprises *μ*_d_, *σ*_d_, *α*_eff_, and *T*_p, 0_. The inversion process is formulated as a least-squares optimization problem, minimizing the non-negative objective function that incorporates both measured values and model-derived estimates of TiRe-LII signals:

(20) $$f_{\mathrm{obj}}={\left\|\frac{b_{\mathrm{mea}}-b_{\mathrm{est}}}{b_{\mathrm{mea}}}\right\|}_{2}^{2}$$

where **b**_mea_ is the measured values of TiRe-LII signals, and **b**_est_ is the model-derived estimates of TiRe-LII signals. 

In a proof-of-concept study, the measured values of TiRe-LII signals are typically replaced by the synthetic LII signal contaminated by a noise model. We employ the general noise model proposed by Sipkens et al. [40] based on extensive experimental data:

(21) $$b_{\mathrm{mea}}=b_{\mathrm{tar}}+\underset{\text{Shot-to-shot error}}{\underset{︸}{\tau \cdot n\cdot b_{\mathrm{tar}}}}+\underset{\mathrm{Poisson}\;\mathrm{error}}{\underset{︸}{{\left[\theta \cdot (1+\tau \cdot n)\cdot b_{\mathrm{tar}}\right]}^{1/2}\circ n^{P}}}+\underset{\mathrm{Gaussian}\;\mathrm{error}}{\underset{︸}{\gamma \cdot n^{G}}}$$

where **b**_tar_ denotes the target (noise-free) simulated TiRe-LII signal, calculated using the full LII model with the true values of variable vector **x**_tar_; *n* is a standard normal random variable; *τ*, *θ*, and *γ* are the characteristic parameters of the general model, representing scale factors for shot-to-shot error, Poisson error, and Gaussian error, respectively; **n**^P^ and **n**^G^ are respectively the standard normal random vector for Poisson error and Gaussian error.

**Figure 2:**
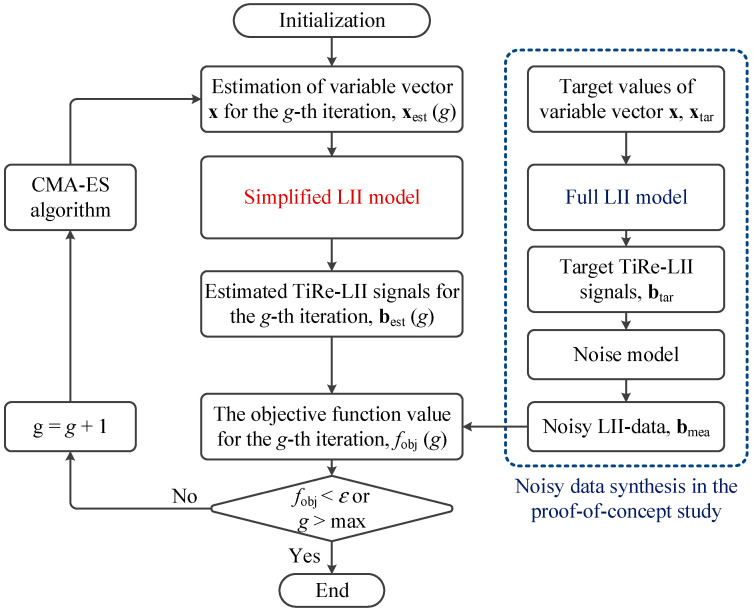
Operation flowchart of the proposed multi-parameter inversion strategy.

To avoid the ‘inverse crime’ phenomenon [41,42], where identical full models and simplified models are used, leading to trivial results, we adopt a dual-model framework, as illustrated in Figure 2. Specifically, the full model generates the synthetic TiRe-LII signal, which differs from the simplified model used for variable estimation. The full and simplified models used in this study are shown in Table 1 (Section 4.2). In this study, we employ the covariance matrix adaptive evolution strategy (CMA-ES) algorithm to minimize the objective function value, with further details available in Ref. [43]. It is worth noting that this study employs the CMA-ES algorithm solely due to its excellent performance demonstrated in previous research. Other non-linear least-square solvers, such as Twomey’s algorithm, could theoretically be applied to this multi-parameter inverse problem, and the performance differences among various algorithms are beyond the scope of this study.

## 4. Results and Discussion

### 4.1. Impact of Flame Temperature Rise on Heat Transfer Sub-Models

In LII technology, rapid pulsed laser heating creates a substantial temperature disparity between the hot particle surface and the relatively cold ambient gas molecules. This initiates various cooling processes for high-temperature particles, including heat conduction, thermionic emission, radiation, sublimation, and oxidative heating. The simplified model selected for the proposed multi-parameter inversion strategy relies on retaining pertinent heat transfer sub-models while excluding trivial ones. This approach ensures modeling accuracy, avoids meaningless calculations, and strikes a balance between computational accuracy and efficiency. Additionally, the chosen simplified model must generalize well to various laser fluences and flame temperatures.

**Figure 3:**
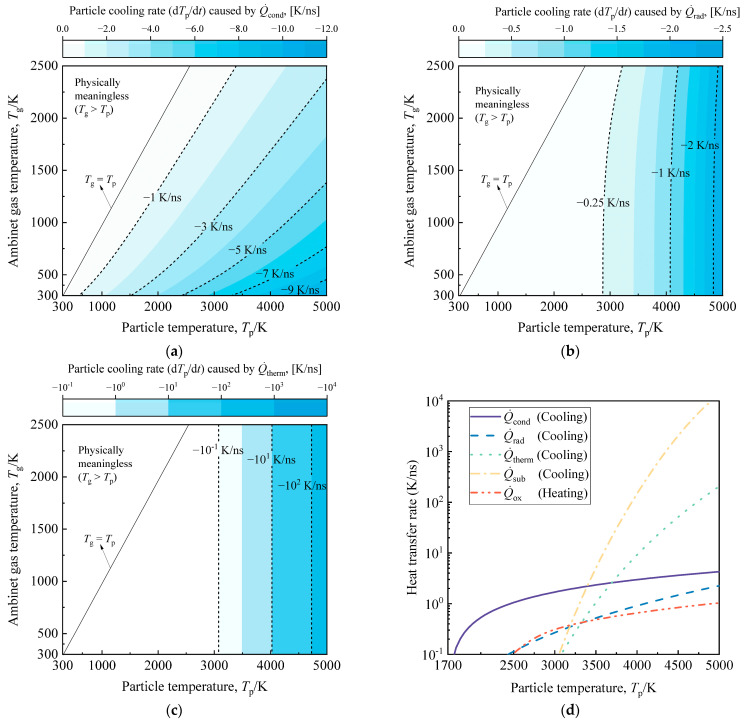
The cooling rates of a typical soot aggregate, with particle temperatures ranging from ambient to 5000 K, due to (**a**) heat conduction, (**b**) radiation, and (**c**) thermionic emission, are investigated as the ambient temperature varies from 300 K to 2500 K. (**d**) Comparison of different heat transfer processes for a typical soot aggregate at various particle temperatures under typical flame conditions (*T*_g_ = 1700 K). The typical soot aggregate studied here has *d*_p_ = 20 nm and *N*_p_ = 30.

**Figure 4:**
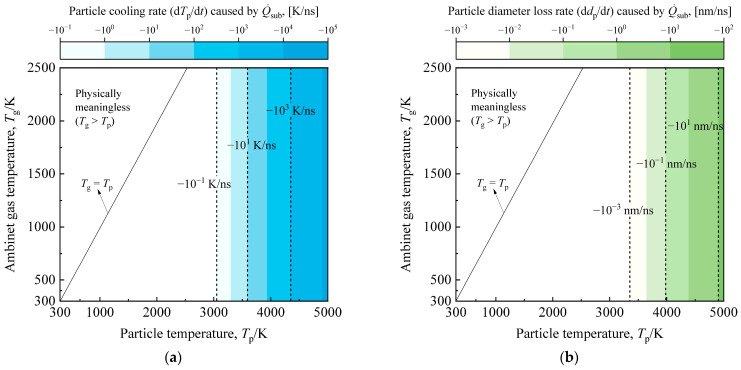
(**a**) The cooling rate and (**b**) particle diameter loss rate of a typical soot aggregate, with particle temperatures ranging from ambient to 5000 K due to sublimation, are investigated as the ambient temperature varies from 300 K to 2500 K. The typical soot aggregate studied here has *d*_p_ = 20 nm and *N*_p_ = 30.

**Figure 5:**
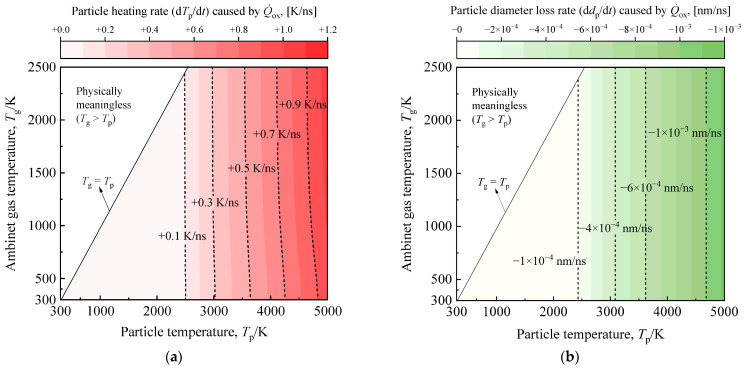
(**a**) The heating rate and (**b**) particle diameter loss rate of a typical soot aggregate, with particle temperatures ranging from ambient to 5000 K due to oxidation, are investigated as the ambient temperature varies from 300 K to 2500 K. The typical soot aggregate studied here has *d*_p_ = 20 nm and *N*_p_ = 30.

To assess the response properties of different heat transfer sub-models for high-temperature soot aggregates to flame temperature rise, we examined the particle heating rate due to oxidation, the particle cooling rates from heat conduction, thermionic emission, radiation, sublimation, and the particle diameter loss rate from sublimation and oxidation. This analysis covered ambient temperatures ranging from 300 K (room temperature) to 2500 K (high flame temperature), as illustrated in Figure 3, Figure 4 and Figure 5. Considering radiation, thermionic emission, sublimation, and oxidation are essentially independent of ambient temperature, Figure 3d illustrates the performance of different heat transfer processes at various particle temperatures under typical flame conditions (*T*_g_ = 1700 K). It is important to note that when particle temperatures exceed 5000 K, soot rapidly evaporates, which is physically unrealistic. Therefore, the temperatures of studied soot in Figure 3, Figure 4 and Figure 5 do not surpass 5000 K. To preserve the soot structure and prevent laser-induced damage, this study recommends selecting a laser fluence not exceeding 0.5 J/cm^2^.

Figure 3a reveals that the conductive cooling rate exhibits a nearly linear increase with the rising particle temperature. Consequently, heat conduction processes consistently drive particle cooling throughout the heating and cooling phases. Moreover, for low laser fluence cases, conduction dominates the soot heat transfer model, as the particle temperature remains below 3500 K, and high-temperature processes are relatively weak (see Figure 3d). Figure 3b illustrates that radiation, although a high-temperature process, has a peak cooling rate below −3 K/ns, making it ignorable. Examining Figure 3c, it becomes evident that thermionic emission is a high-temperature process independent of ambient temperature, contributing negligibly to particle cooling when the particle temperature is under 3500 K (see Figure 3d). Nevertheless, as the particle temperature increases, the thermionic emission cooling rate exhibits exponential growth, surpassing 100 K/ns. The duration of the high-temperature phase, where the particle temperature exceeds 3500 K (see Figure 3d), becomes crucial in deciding whether to include the thermionic emission term in the simplified model.

It is essential to note the exponential response of sublimation to the rise in particle temperature, as shown in Figure 4. For particles with temperatures between 4000 K and 5000 K, the soot aggregate cooling rates due to sublimation range from −10^2^ K/ns to −10^4^ K/ns, accompanied by particle size loss ranging from −0.1 nm/ns to −10 nm/ns. Thus, when particles undergo the same high-temperature phase, the change in particle energy due to sublimation becomes more crucial than thermionic emission, and the alterations in particle mass and diameter due to sublimation cannot be overlooked. For the proposed multi-parameter inversion strategy, both sublimation and thermionic emission play pivotal roles in modeling in-flame soot TiRe-LII. Sublimation’s role must be considered, while thermionic emission relies on the chosen laser fluence. Thermionic emission-induced particle cooling, with higher laser fluences, may briefly surpass the sublimation process, but accuracy in modeling both becomes problematic in extreme high-temperature cases. Consequently, the sublimation term consistently proves stronger than thermionic emission within the range of laser fluences considered in this study.

Similarly, the oxidation of high-temperature particles induces changes in both energy and mass within the soot. However, as illustrated in Figure 5, the oxidative heating rate of high-temperature soot does not exceed 1.5 K/ns. Furthermore, the particle size loss due to oxidation is less pronounced than anticipated, remaining below 0.001 nm/ns. In instances of extremely high temperatures induced by elevated laser fluences, the heat and mass transfer model for the oxidation term encounters significant errors. Consequently, in the context of this study, where model accuracy takes precedence, disregarding the heating of soot due to oxidation and the associated mass consumption is deemed acceptable. Therefore, the impact of the oxidation process on the modeling of in-flame soot TiRe-LII can be considered negligible. Investigation of the response characteristics of different heat transfer mechanisms as particle temperature rises under a wide range of ambient temperature conditions from 300 K to 2500 K has been conducted above. It is noteworthy that similar studies have been extensively discussed in prior research [10,34], with trends consistent with those presented in this paper being observed.

### 4.2. Simplified LII Model for Multi-Parameter Inversion

In light of the insights gained from the analysis in Section 4.1, where distinct responses of various heat transfer sub-models to ambient temperature variations were observed, it becomes evident that choosing a suitable simplified LII model for multi-parameter inversion necessitates a quantitative evaluation of model errors. As outlined in Table 1, the full model (referred to as Model #0), encompassing all conceivable heat transfer sub-models, serves as the baseline for assessing model accuracy. This model incorporates internal energy, laser energy absorption, heat conduction, radiation, sublimation, thermionic emission, and oxidation terms in the energy balance equation, alongside sublimation and oxidation terms in the mass balance equation.

In the initial assessment, the applicability of a widely used low-laser fluence model (designated as Model #1) is examined. Model #1 retains only the internal energy, laser energy absorption, and heat transfer terms in its energy balance model, omitting all mass losses. Employing a laser fluence of 0.12 J/cm^2^, the performance of the low-laser fluence model (Model #1) is compared with the full model (Model #0, baseline) in reproducing soot temperature and TiRe-LII signal profiles under three typical ambient temperature conditions: 300 K (room temperature), 1100 K (low flame temperature), and 1700 K (high flame temperature). 

**Figure 6:**
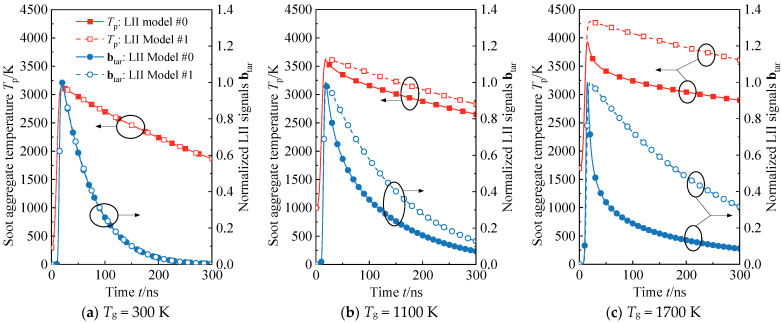
Comparison of Model #0 (full model, baseline) and Model #1 (low-fluence model) in reproducing particle temperature and TiRe-LII signal profiles when ambient temperatures are (**a**) 300 K, (**b**) 1100 K, and (**c**) 1700 K, respectively. The curves indicated by the leftward black arrow in the figure corresponds to the left y-axis, while the curves indicated by the rightward black arrow corresponds to the right y-axis.

Under room temperature conditions, the particle temperature and TiRe-LII signal curves reproduced by Model #1 align perfectly with those of the baseline model, indicating negligible model error at this point (refer to Figure 6a). However, in the low-temperature region of the flame, the particle temperature profile generated by Model #1 diverges from the baseline model after the peak temperature, maintaining an elevated value of approximately 300 K. This results in an error exceeding 50% in the TiRe-LII signal reproduced by the spectroscopy model (see Figure 6b). The modeling error of Model #1, relative to the baseline model, becomes more pronounced with an increase in soot ambient temperature to high flame temperatures. In comparison with the baseline model, the peak moment of the particle temperature profile is significantly delayed, and the cooling trend after the peak temperature differs entirely. Model #1 appears to neglect certain crucial cooling processes, leading to an overall higher particle temperature of about 800 K in the cooling phase compared to the baseline model. At this point, the error in the TiRe-LII signal curve of Model #1 exceeds 100%, with curve characteristics inconsistent with the baseline model (see Figure 6c). It is noteworthy that these TiRe-LII models have been extensively discussed and compared in detail in References [10,34]. Their evaluations yield results consistent with those presented in this paper, providing additional support for the subsequent model selection in our study.

Given the inadequacy of the low-fluence model under flame temperature conditions, a particular simplified model (referred to as Model #2) based on Model #1 is chosen for the in-flame soot multi-parameter inversion strategy in this study. In comparison to the full model (Model #0), Model #2 essentially neglects weak heat transfer mechanisms, including oxidation and annealing. Model #2 retains the sublimation and thermionic emission terms in the energy balance equation, and the mass balance equation includes the sublimation term, as indicated in Table 1. The temperature gradient in the region adjacent to the peak temperature is substantial, and a weak model bias can cause the reproduced particle temperature profile to deviate at the peak temperature and introduce a time delay. Therefore, the moment when the particle reaches the peak temperature, *t*_m_ serves as an intuitive indicator for model error assessment. 

**Figure 7:**
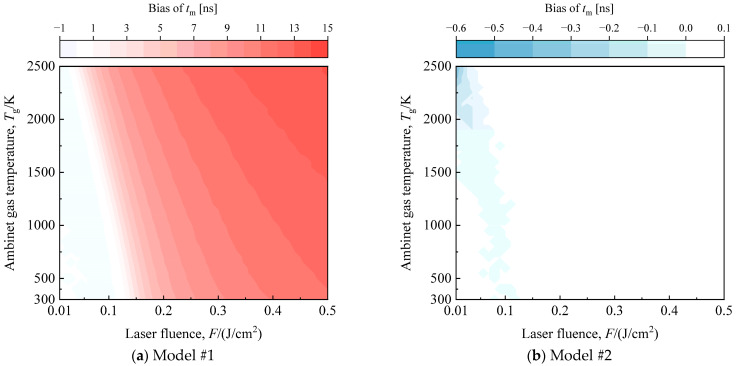
Comparison of the bias of (**a**) Model #1 (low-fluence model) and (**b**) Model #2 (simplified LII model for multi-parameter inversion strategy) with respect to the baseline model at the peak temperature moment (*t*_m_) for different laser fluence and ambient temperatures.

Comparing the deviations in *t*_m_ for Model #2 and Model #1 relative to the baseline model under different laser energies and ambient temperatures (Figure 7), it is evident that, even with a lower laser fluence, Model #1 is only suitable for limited conditions around room temperature. Once the ambient temperature is increased or the laser fluence is elevated, the peak particle profile reproduced by Model #1 experiences a significant time delay. In contrast, Model #2, accounting for sublimation and hot electron emission contributions, demonstrates excellent generalization. Figure 7b illustrates that the error of Model #2 relative to the baseline model is negligible across a wide temperature range, from room temperature to high flame temperature, as long as the laser fluence exceeds 0.1 J/cm^2^. Consequently, all models used in the subsequent studies in this research adhere to Model #2.

### 4.3. Impact of Flame Temperature Bias on LII-Based Particle Sizing

In conventional LII-based particle sizing, only two parameters of the particle size distribution, namely the mean *μ*_d_ and the standard deviation *σ*_d_ of the log-normal distribution, are treated as unknown variables for inversion. The flame temperature is typically a predetermined parameter in the LII model for this binary inverse problem. It is worth noting that in recent years, an increasing number of studies have treated flame temperature as a stochastic variable with an expectation value and uncertainty [23]. In this analysis, the flame temperature is assumed to have varying degrees of deviation to examine the perturbation of flame temperature bias on LII-based particle sizing. 

The normalized noisy TiRe-LII signal **b**_mea_ used for inverting *μ*_d_ and *σ*_d_ is mimicked by the full model along with the noise model (refer to Section 3.2). The temporal profile of the selected pulsed laser approximates a Gaussian distribution *g*(*t*):

(22) $$g\left(t\right)=\frac{1}{\sqrt{2\pi }\sigma_{\mathrm{Laser}\;}}\exp\left[-\frac{{\left(t-\mu_{\mathrm{Laser}}\right)}^{2}}{2\sigma_{\mathrm{Laser}}^{2}}\right]$$

where *μ*_Laser_ = 22.5 ns and *σ*_Laser_ = 3.3 ns are the mean and standard deviation of the *g*(*t*), respectively. 

*q*(*t*) is the temporal profile of the normalized laser energy:

(23) $$q\left(t\right)=g\left(t\right)/\int g\left(t^{\prime }\right)dt^{\prime }$$

Based on these settings, the laser energy absorption of soot aggregates is determined by the laser fluence *F* by [31]:

(24) $${\dot{Q}}_{\mathrm{abs}}\left(t\right)=C_{\mathrm{abs},\;\lambda }\left(d_{p},\;m_{\lambda }\right)F\cdot q\left(t\right)$$

**Figure 8:**
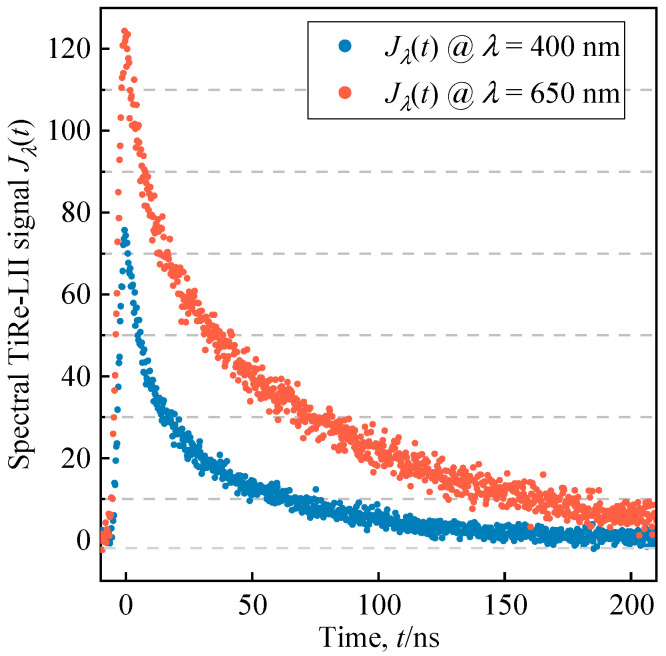
Example of a set of two-color noisy spectral TiRe-LII signals generated by the coupling of the full model and the general noise model for a binary inversion problem in which two particle size distribution parameters are inverted simultaneously.

Substituting the above-pulsed laser settings into the full model produces the smooth and noise-free TiRe-LII, **b**_tar_. Subsequently, a noisy TiRe-LII **b**_mea_, similar to the real measured signal, is obtained from **b**_tar_ through the noise model. Figure 8 illustrates an example of a set of two-color noisy spectral TiRe-LII signals (signals prior to normalization, thus denoted as *J_λ_*(*t*)). Because the noise model mimics the irregular perturbations of real measurement noise through random numbers, each set of **b**_mea_ generated through the noise model is different. 

For a pulsed laser with fluence *F* = 0.12 J/cm^2^ and wavelength of 532 nm, and the true value of flame temperature *T*_g_ of 1700 K, 100 sets of different **b**_mea_ are generated for the binary inverse problem of the mean (*μ*_d_) and standard deviation (*σ*_d_) of the log-normal distribution representing soot primary particle sizes. It is important to note that in this binary inverse problem, the true value for *μ*_d_ is 20 nm, and the true value for *σ*_d_ is 1.2. Thirteen cases with different *T*_g_ biases are considered, ranging from prior values of *T*_g_ of 1500 K to 1900 K, and the corresponding percentages of *T*_g_-Bias range from −11.8% to +11.8%, as shown in Table 2.

**Figure 9:**
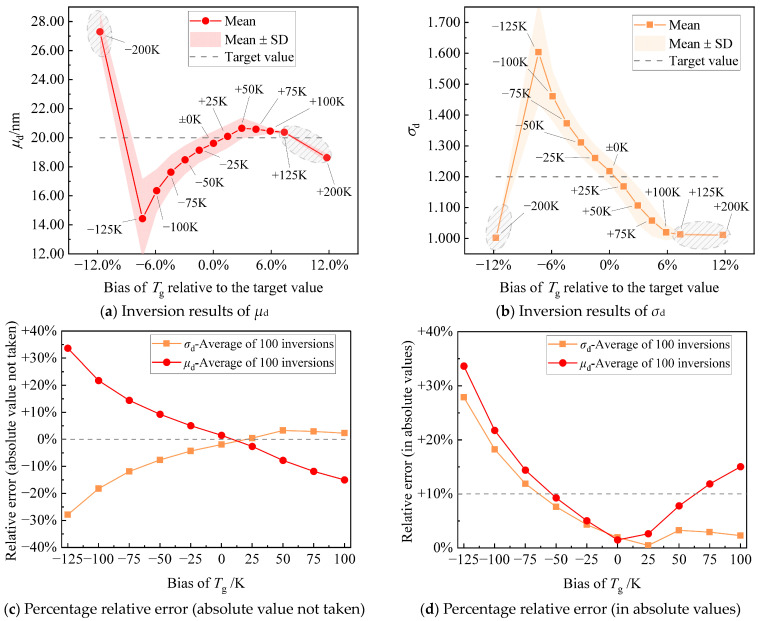
Results of 100 inversions of (**a**) *μ*_d_ and (**b**) *σ*_d_ for thirteen cases under different *T*_g_ biases; the square-dashed line in the figure is the target value of the parameter to be inverted; the circle-solid line is the average of the 100 inversion results; the text next to the circle labels the bias of *T*_g_; the shaded area of the coloring is the confidence interval of one standard deviation; and shaded circles denote three potential artifact cases. After excluding three potential artifact cases, the percentage relative error of the average of 100 inversions is presented for (**c**) without taking absolute values and (**d**) after taking absolute values, showing variations with *T*_g_ biases. The reference line at 0% on the y-axis in (**c**) serves as a baseline for the negative correlation between *μ*_d_ and *σ*_d_. The reference line at 10% on the y-axis in (**d**) serves as a benchmark for the satisfactory error level.

Figure 9a,b summarize the results of 100 inversions of *μ*_d_ and *σ*_d_ for thirteen cases at different *T*_g_ biases, respectively. When underestimating the flame temperature, as the *T*_g_ bias increases from −25 K to −125 K, the distribution of resulting *μ*_d_ and *σ*_d_ essentially widens. This suggests a decrease in the credibility of the solution, posing a challenge in obtaining stable inversion results. However, as the *T*_g_ bias further increases to −200 K, the confidence for the 100 inversions of *μ*_d_ and *σ*_d_ narrows unexpectedly. It is crucial to note that, in this case, the resulting *μ*_d_ and *σ*_d_ from the 100 inversions are higher and lower than the target value, respectively, which contrasts with the trend observed between *T*_g_ biases of −25 K and −125 K. This anomaly arises from *σ*_d_ in the binary inverse problem. As illustrated in Figure 9b, when the *T*_g_ bias is −200 K, the 100 inversions for *σ*_d_ are all trapped at *σ*_d_ = 1, the boundary of the solution space. The correlation matrix analysis of Bauer et al. [23] shows that there is a negative correlation between *μ*_d_ and *σ*_d_. Given this, when the solution of *σ*_d_ is trapped at one below the target value, this, in turn, causes the solution of *μ*_d_ to be limited to values greater than the target value. It is evident that when *T*_g_ bias exceeds −200 K, the binary inverse problem becomes incapable of deriving a global optimal solution for *μ*_d_ and *σ*_d_, rendering the inversion results unreliable. Therefore, the simulations with a *T*_g_ bias of −200 K in Figure 9a,b are potential artifacts (marked by shaded circles). When overestimating the flame temperature, as the *T*_g_ bias increases from +25 K to +100 K, the distribution width of resulting *μ*_d_ and *σ*_d_ remains essentially unchanged. However, as *T*_g_ bias further increases to +125 K and +200 K, the confidence intervals for *μ*_d_ and *σ*_d_ suddenly narrow, which is attributed to *σ*_d_ becoming trapped at the solution space boundary. Therefore, the simulations with a *T*_g_ bias of +125 K and +200 K in Figure 9a,b are also potential artifacts (marked by shaded circles). To precisely understand the impact of flame temperature bias on the inversion result errors, after excluding the three aforementioned artifact cases, the percentage relative error of the average of 100 inversions is illustrated both without taking absolute values and after taking absolute values. These variations with *T*_g_ biases are presented in Figure 9c,d, respectively. The relative errors of *μ*_d_ and *σ*_d_ in Figure 9c basically show a monotonically increasing trend with respect to the *T*_g_ biases. The slope for *μ*_d_ is negative, while for *σ*_d_, it is positive, aligning with the expected negative correlation between the two. In Figure 9d, after taking the absolute values of the relative errors of *μ*_d_ and *σ*_d_, both *μ*_d_ and *σ*_d_ show a U-shaped function with respect to the *T*_g_ biases. From Figure 9d, it is evident that LII-based particle sizing is sensitive to flame temperature bias. To obtain satisfactory results for both *μ*_d_ and *σ*_d_ (with relative errors <±10%), the tolerable bias in the prior flame temperature is approximately within the range of ±50 K. In light of this, when the independent measurement error of flame temperature is within ±50 K (achievable through various methods, including thermocouple [44], laser absorption spectroscopy [45], and spectral soot emission-based two-color pyrometry [46]), it is still preferable to treat LII-based particle sizing of in-flame soot as a binary inverse problem. Due to the ill-posed nature of inverse problems, simultaneously inverting fewer parameters aids in obtaining solutions with higher confidence and precision. However, in the absence of supplementary measurement techniques or reliable prior knowledge of flame temperature, independently using LII for in-flame soot particle sizing involves a multi-parameter inverse problem that includes both particle size distribution and flame temperature. 

### 4.4. Multi-Parameter Inversions at Two Typical Flame Temperatures

**Figure 10:**
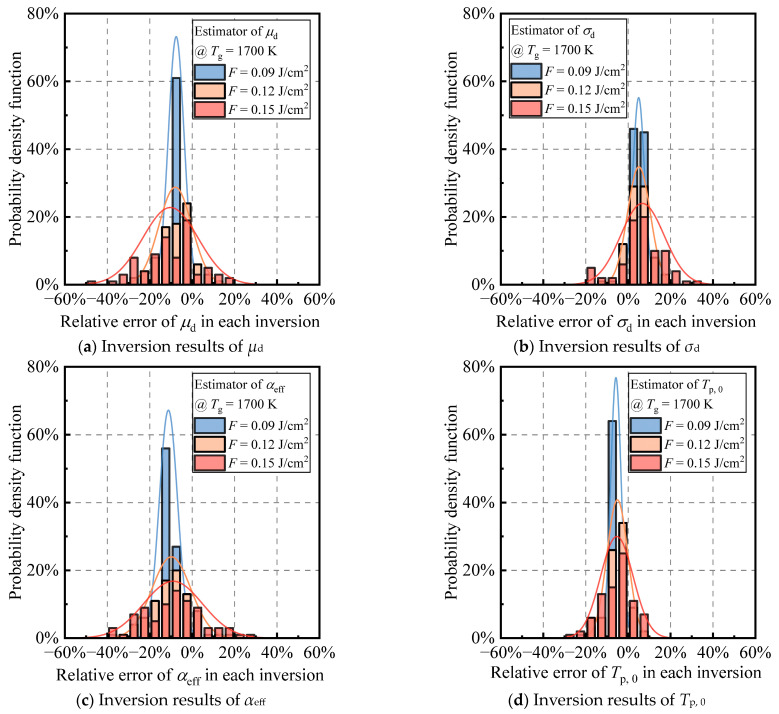
Histograms of the relative error per inversion for 100 multi-parameter joint inversions of (**a**) *μ*_d_, (**b**) *σ*_d_, (**c**) *α*_eff_, and (**d**) *T*_p, 0_ (=*T*_g_) using the laser fluence of 0.09 J/cm^2^, 0.12 J/cm^2^, and 0.15 J/cm^2^, respectively, when the studied soot aggregates were bathed in a flame temperature of 1700 K. This figure is based on Figure A1, excluding the unreasonable four-parameter inversion results at the *σ*_d_ = 1 peak.

After evaluating the distinct responses of various heat transfer mechanisms to the temperature increase of ambient gases and laser-heated particles (Section 4.1), we selected a specific simplified LII model (Section 4.2). This model incorporates sublimation and thermionic emission in addition to the low-fluence model. In scenarios where supplementary temperature measurement methods are unavailable and reliable prior information on flame temperature is lacking, there is a need to enhance the independent use of LII technology (Section 4.3). Therefore, based on this simplified LII model, this study proposes a multi-parameter inversion strategy using only the TiRe-LII signals. The strategy involves the simultaneous inversion of the equivalent thermal accommodation coefficient (*α*_eff_) and the flame temperature (*T*_g_, equal to the initial temperature of the particles prior to laser heating, *T*_p, 0_), along with the two-particle size distribution parameters, *μ*_d_ and *σ*_d_, which is essentially a four-variable inverse problem based on normalized noisy TiR-LII signals. Three laser fluences, *F* = 0.09 J/cm^2^, *F* = 0.12 J/cm^2^, and *F* = 0.15 J/cm^2^ are used to generate 100 sets of different **b**_mea_ for studying the multi-parameter inversion strategy. On this basis, the performance of the multi-parameter inversion strategy was first evaluated for a typical flame temperature of 1700 K. The relative errors per inversion for 100 multi-parameter joint inversions are summarized as histograms in Figure 10.

The relative errors of each inversion of the four target parameters under different laser fluences are approximated to show a single-peaked normal distribution, indicating that the results of 100 inversions tend to converge to the same solution. In terms of the distribution width, it not only indicates inversion stability but also reflects the extent to which noise is amplified into the solution and the credibility of the obtained results. The distribution of the 100 inversion results for all four parameters becomes wider and wider with the increase of the laser energy under the flame temperature of 1700 K, indicating that the inversion stability deteriorates. This phenomenon could be explained as follows: Among the four inversion parameters, α_eff_ plays a pivotal role in governing the heat conduction mechanism. A stronger heat conduction mechanism implies more robust constraints on α_eff_ during inversion, yielding more accurate results. Additionally, according to Bauer et al.’s correlation matrix analysis [23], there is a significant correlation (0.49) between *μ*_d_ and α_eff_. At a flame temperature of 1700 K, employing a laser fluence of 0.09 J/cm^2^ achieves a balance between the sublimation and heat conduction mechanisms during particle cooling. This balance ensures precise inversion results for α_eff_, and the mathematical constraints imposed by the sublimation mechanism on *μ*_d_ enhance the accuracy of its inversion. In turn, this enhances the inversion accuracy of α_eff_. The accurate inversion of *μ*_d_, in turn, leads to a corresponding accuracy in retrieving *σ*_d_. Considering the strong correlation (correlation coefficient of 0.62) between *σ*_d_ and *T*_g_, this interrelation significantly influences the *T*_g_ inversion process, ensuring simultaneous precision in *T*_g_ inversion. However, with an increase in laser fluence, heat conduction experiences approximately linear growth, while sublimation undergoes exponential increase. This disruption in balance alters the above inversion constraint relationship, causing a relative decrease in the contribution of the heat conduction mechanism to particle cooling. Consequently, the precision of α_eff_ inversion diminishes, and the uncertainty of *μ*_d_ inversion increases. The diminished precision in both inversions is transmitted through their correlation, resulting in an overall amplification of the inversion error and uncertainty for all four parameters (see Table 3). Therefore, a suitable laser fluence needs to be selected to ensure that the multi-parameter inversion strategy can give a stable and accurate inversion of the particle size distribution and flame temperature. At this point, for the high flame temperature case of 1700 K, 0.09 J/cm^2^ is the best choice. In this case, the average of 100 inversions is close to the target values of the four parameters, and the average relative errors of 100 inversions for the four parameters are less than 10%, which has excellent inversion accuracy.

Under the laser fluence of 0.09 J/cm^2^, the multi-parameter inversion strategy employed in this study yields an average flame temperature error of 75 K, with an average uncertainty of 42 K (see Table 3). In terms of accuracy, there is a certain gap compared to the commonly used temperature measurement techniques (such as thermocouple, laser absorption spectroscopy, and two-color pyrometry) with errors ranging from 50 to 60 K [44], while uncertainty levels are comparable. For scenarios focused on in this study, where flame temperature measurement tools are unavailable and reliable prior information about the flame is lacking, reasonably accurate and uncertain flame temperature information can be obtained solely using LII signals. However, to achieve higher precision, the combination of LII with supplementary temperature measurement techniques is undoubtedly a superior choice, whether for flame temperature or particle size distribution. Due to the ill-posed nature of inverse problems, reducing the number of inversion parameters reduces the errors and uncertainties in the inversion of particle size distribution. Furthermore, it is essential to emphasize that the limitations of this study lie in the fact that, apart from the four target parameters for inversion, the remaining model parameters, such as *E*(*m*), soot density, and work function, are treated as fixed values and assumed to be perfectly known. Therefore, the inversion results in Figure 10 are obtained under idealized conditions. According to Bauer et al.’s findings, uncertainties in model parameters are likely to broaden the existing distribution of inversion results, reducing confidence. 

**Figure 11:**
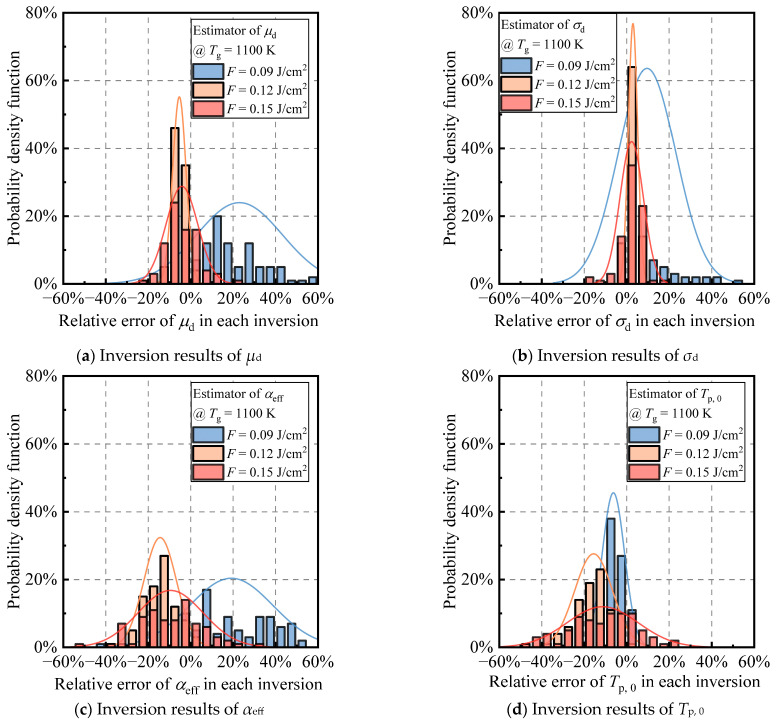
Histograms of the relative error per inversion for 100 multi-parameter joint inversions of (**a**) *μ*_d_, (**b**) *σ*_d_, (**c**) *α*_eff_, and (**d**) *T*_p, 0_ (=*T*_g_) using the laser fluence of 0.09 J/cm^2^, 0.12 J/cm^2^, and 0.15 J/cm^2^, respectively, when the studied soot aggregates were bathed in a flame temperature of 1100 K. This figure is based on Figure A2, excluding the unreasonable four-parameter inversion results at the *σ*_d_ = 1 peak.

To evaluate the performance of the proposed multi-parameter inversion strategy at different flame temperatures, a relatively low flame temperature of 1100 K is chosen to carry out the same inversion accuracy and stability evaluation as in Figure 10, and the corresponding results are summarized in Figure 11. Although the overall distribution of 100 inversions still approximately shows a single-peaked normal distribution, the distribution is wider, and the central axis of the distribution has significantly deviated from the target value in some cases, which indicates that the stability and accuracy of the inversions have declined to different degrees. In the 100 multi-parameter inversion results at 0.09 J/cm^2^ laser fluence, the average error of both particle size distribution parameters exceeds 20%, and the average relative error of flame temperature exceeds 10%, which does not meet the practical requirements. The 100 multi-parameter inversion results for 0.12 J/cm^2^ and 0.15 J/cm^2^ perform better, with the average errors of the four parameters controlled within 10%, but the standard deviation of the inversion in the latter case is larger overall. Therefore, considering the accuracy and stability of the inversion, 0.12 J/cm^2^ is the best choice for the multi-parameter inversion strategy at 1100 K flame temperature. The overall accuracy and reliability of the multi-parameter inversion strategy decreases when compared to the flame temperature of 1700 K (see Table 3), and therefore, the proposed method is more suitable for higher flame temperatures, such as 1700 K. 

**Figure A1:**
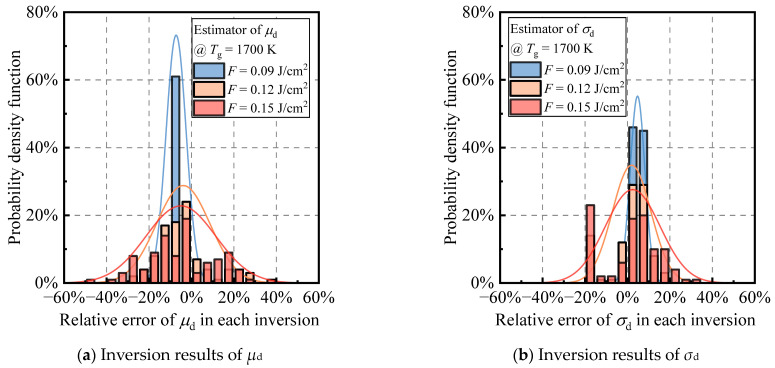

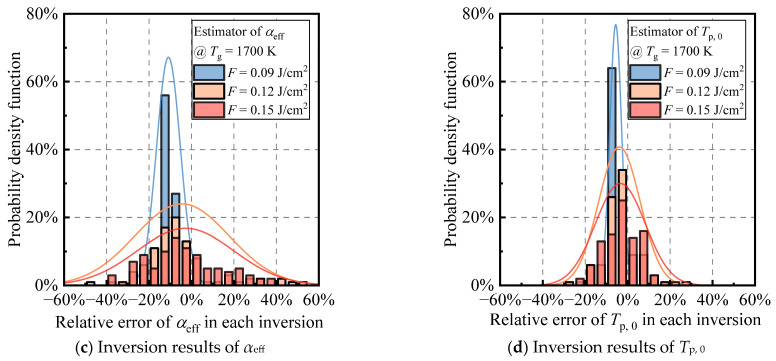
Histograms of the relative error per inversion for 100 multi-parameter joint inversions of (**a**) *μ*_d_, (**b**) *σ*_d_, (**c**) *α*_eff_, and (**d**) *T*_p, 0_ (=*T*_g_) using the laser fluence of 0.09 J/cm^2^, 0.12 J/cm^2^, and 0.15 J/cm^2^, respectively, when the studied soot aggregates were bathed in a flame temperature of 1700 K.

**Figure A2:**
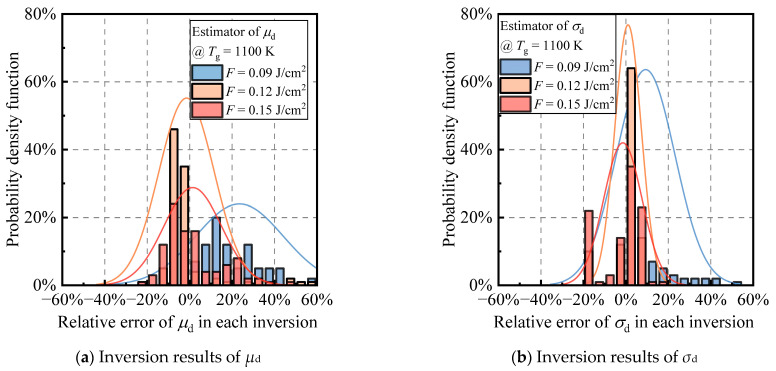

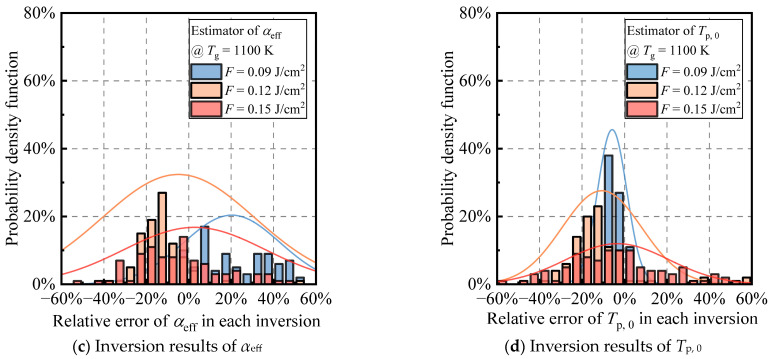
Histograms of the relative error per inversion for 100 multi-parameter joint inversions of (**a**) *μ*_d_, (**b**) *σ*_d_, (**c**) *α*_eff_, and (**d**) *T*_p, 0_ (=*T*_g_) using the laser fluence of 0.09 J/cm^2^, 0.12 J/cm^2^, and 0.15 J/cm^2^, respectively, when the studied soot aggregates were bathed in a flame temperature of 1100 K.

It is noteworthy that as the flame temperature decreases from 1700 K to 1100 K, the optimal laser fluence for the multi-parameter inversion strategy shifts from 0.09 J/cm^2^ to a higher 0.12 J/cm^2^. The inversion accuracy and stability at 0.09 J/cm^2^ become the worst among the three laser fluences. This shift is attributed to the shorter high-temperature segment of the particle temperature and overall heat conduction dominating the primary heat transfer model. Previous studies have revealed undesirable mathematical properties in inversion parameters using conduction-dominated LII signals, contributing to a significant ill-posedness in the multi-parameter inverse problem [23,47]. Consequently, careful selection of laser fluence is crucial for multi-parameter inversion strategies at different flame temperatures, with the optimal laser fluence correlating with the flame temperature. Another point worth mentioning is that similar to Figure 9, the results of the four-parameter inversions in Figure 10 and Figure 11 are also affected by artifacts at *σ*_d_ = 1. Figure A1, Figure A2, and Table A1 represent the situations in which the results at the *σ*_d_ = 1 peak were not excluded from Figure 10, Figure 11, and Table 3, respectively. It is evident that the distributions in Figure A1b and Figure A2b are not strictly unimodal, exhibiting a secondary peak at *σ*_d_ = 1, especially under the high laser fluence of 0.15 J/cm^2^. Due to this influence, the distributions of the 100 inversions for the four parameters become broader, as intuitively reflected in Table A1, where the standard deviations of the inversion results are higher than those in Table 3. This leads to an increase in the uncertainty of the inversion results. Additionally, influenced by the negative correlation between *μ*_d_ and *σ*_d_ (with a correlation coefficient of approximately −1), Figure A1a and Figure A2a both exhibit a peak in *μ*_d_ relative error at +20%, as the *σ*_d_ = 1 peak is located at a relative error of −20%. Considering that the inversion results at *σ*_d_ = 1 are artifacts caused by local optimal points, excluding these results is reasonable. Through this processing step, there has been a moderate improvement in both the confidence and accuracy of the inversion results.

Overall, with the careful selection of laser fluence, the multi-parameter inversion strategy presented in this study demonstrates the capability to simultaneously extract particle size distribution parameters, flame temperature, and thermal accommodation coefficient from TiRe-LII signals. While accurate inversion results for particle size distribution parameters can be achieved, the flame temperature results exhibit relatively high uncertainties, ranging from 100 K to 150 K.

## 5. Conclusions

Time-resolved laser-induced incandescence (TiRe-LII) emerges as a powerful non-contact diagnostic technology for particle sizing of in-flame soot aggregates. However, it typically requires independent methods for pre-measuring flame temperature. In scenarios where there are no available supplementary measurement methods or reliable prior knowledge of flame temperature, LII-based particle sizing cannot be conducted for soot in flames. To address such situations, this study proposes a multi-parameter inversion strategy solely utilizing TiRe-LII signals. This strategy simultaneously inverts four parameters: the mean (*μ*_d_) and standard deviation (*σ*_d_) of soot primary particle size distribution, flame temperature *T*_g_, and equivalent thermal accommodation coefficient *α*_eff_. The main conclusions are as follows:

(1) The numerical investigation explores the sensitivity of LII-based bivariate inversion of *μ*_d_ and *σ*_d_ to flame temperature bias. Even slight flame temperature bias (−100 K) led to inversion errors exceeding 20%. Conventional bivariate inversion of *μ*_d_ and *σ*_d_ is recommended when combining LII with other measurement methods featuring a *T*_g_ uncertainty of ±50 K. However, it becomes challenging in the absence of supplementary measurement methods or reliable prior knowledge of flame temperature.

(2) By employing a reduced laser fluence of 0.09 J/cm^2^, the multi-parameter inversion strategy proposed in this study yields precise inversion results for the two-particle size distribution parameters and offers flame temperature estimates with uncertainties ranging from 100 K to 150 K at a flame temperature of 1700 K. However, at a flame temperature of 1100 K, the overall precision and confidence of the four-parameter inversion decrease, suggesting that the strategy is more apt for higher flame temperatures.

In conclusion, with careful selection of laser fluence, the multi-parameter inversion strategy proposed in this study enables the simultaneous inversion of *μ*_d_, *σ*_d_, *T*_g_, and *α*_eff_ solely from TiRe-LII signals without relying on independent temperature measurement or prior knowledge of flame temperature. Although the precision of flame temperature obtained through this approach (approximately 100 K) shows some disparity compared to specialized flame temperature measurement techniques (50 K~60 K), it nonetheless makes it feasible to independently use LII technology for particle sizing in high-temperature environments. 

For future work, incorporating the boundary sphere-based Fuchs heat conduction model and the annealing model proposed by Michelson [31] into the LII model can increase the disparity between the full and simplified models, aiding in the comprehensive assessment of the multi-parameter inversion strategy. Furthermore, employing Bayesian estimation and treating the remaining model parameters, such as laser fluence, *E*(*m*), soot density, and work function, as variables with expectation and uncertainties is crucial for evaluating the impact of their prior information uncertainties on the performance of the multi-parameter inversion strategy.

## Figures and Tables

**Table 1:** Three TiRe-LII models were involved in this study.

Notation	Type	Energy and Mass Balance Equations
Model #0	Baseline (Full model)	$\begin{array}{c} \dot{U}={\dot{Q}}_{\mathrm{abs}}+{\dot{Q}}_{\mathrm{cond}}+{\dot{Q}}_{\mathrm{rad}}+{\dot{Q}}_{\mathrm{sub}}+{\dot{Q}}_{\mathrm{therm}}+{\dot{Q}}_{\mathrm{ox}}\\ \dot{M}={\dot{M}}_{\mathrm{sub}}+{\dot{M}}_{\mathrm{ox}} \end{array}$
Model #1	Low-fluence model (Simplified model)	$\begin{array}{c} \dot{U}={\dot{Q}}_{\mathrm{abs}}+{\dot{Q}}_{\mathrm{cond}}\\ \dot{M}=0 \end{array}$
Model #2	Selected for the multi-parameter inversion in this study (Simplified model)	$\begin{array}{c} \dot{U}={\dot{Q}}_{\mathrm{abs}}+{\dot{Q}}_{\mathrm{cond}}+{\dot{Q}}_{\mathrm{sub}}+{\dot{Q}}_{\mathrm{therm}}\\ \dot{M}={\dot{M}}_{\mathrm{sub}}+{\dot{M}}_{\mathrm{ox}} \end{array}$

**Table 2:** Flame temperature bias setting for studying the binary inverse problem of *μ*_d_ and *σ*_d_.

Prior Value of *T*_g_	1700 K	1725 K/ 1675 K	1750 K/ 1650 K	1775 K/ 1625 K	1800 K/ 1600 K	1825 K/ 1575 K	1900 K/ 1500 K
Bias of *T*_g_	0 K	±25 K	±50 K	±75 K	±100 K	±125 K	±200 K
Percentage of *T*_g_-Bias	0%	±1.5%	+2.9%	±4.4%	±5.9%	+7.4%	+11.8%

**Table 3:** Four-parameter inversion results were obtained using three laser fluences when the flame temperatures were 1700 K and 1100 K, respectively. The results presented in this table are based on Table A1, excluding the unreasonable four-parameter inversion results at the *σ*_d_ = 1 peak.

Scenario	*F* = 0.09 J/cm^2^	*F* = 0.12 J/cm^2^	*F* = 0.15 J/cm^2^
*T*_g_ = 1700 K (Corresponding to Figure 10)			
*μ*_d_/nm	18.88 ± 0.74	18.40 ± 1.55	17.87 ± 2.55
*σ*_d_	1.25 ± 0.03	1.23 ± 0.15	1.28 ± 0.12
α_eff_	0.24 ± 0.01	0.23 ± 0.02	0.23 ± 0.03
*T*_g_/K	1625 ± 42	1613 ± 84	1605 ± 128
*T*_g_ = 1100 K (Corresponding to Figure 11)			
*μ*_d_/nm	24.31 ± 4.03	19.09 ± 0.63	19.17 ± 1.51
*σ*_d_	1.28 ± 0.16	1.23 ± 0.03	1.23 ± 0.07
α_eff_	0.32 ± 0.05	0.22 ± 0.02	0.24 ± 0.04
*T*_g_/K	1040 ± 58	937 ± 98	975 ± 185

## Data Availability

The data presented in this study are available on request from the corresponding author. The data are not publicly available due to privacy.

## Appendix A

### Appendix A.1. Simulation of Normalized TiRe-LII Signal Considering Both the Size Polydispersity of Soot Primary Particles and Aggregates

For optically thin soot aggregate aerosols with polydisperse primary particles diameter *d*_p_, the spectral TiRe-LII signals emitted by laser-heated soot at an instant *t*, denoted as *J_λ_*(*t*), is calculated as follows:

(A1)
$$J_{\lambda }\left(t\right)=\Lambda \int p\left(d_{p}\right)C_{\mathrm{abs},\;\lambda }\left[d_{p}\left(t\right)\right]I_{b,\;\lambda }\left[T_{p}\left(t\right)\right]d\left(d_{p}\right)$$

where Λ is the intensity scaling factor, accounting for the effect of soot volume fraction, geometry, and collection efficiency of detectors; *p*(*d*_p_) is the probability density function of the polydisperse primary particles of soot aggregates; *C*_abs, *λ*_ is the absorption cross-section of soot aggregates at wavelength *λ*, which depends on the *d*_p_; *I*_b, *λ*_ is the blackbody spectral intensity emitted by the soot aggregates at temperature *T*_p_.

Building upon Equation (A1) and simultaneously considering the polydispersity in sizes *N*_p_ among different aggregates, the spectral TiRe-LII signals of a group of laser-heated soot aggregates can be expressed as:

(A2)
$$\begin{array}{cc} S\left(\lambda ,\;t\right) & =\int p\left(N_{p}\right)N_{p}J_{\lambda }\left(t\right)d\left(N_{p}\right)\\ & =\Lambda \iint p\left(N_{p}\right)p\left(d_{p}\right)N_{p}C_{\mathrm{abs},\;\lambda }\left[d_{p}\left(t\right)\right]I_{b,\;\lambda }\left[T_{p}\left(t\right)\right]d\left(d_{p}\right)d\left(N_{p}\right) \end{array}$$

where *p*(*N*_p_) is the probability density function of aggregate size *N*_p_.

Then, the spectral TiRe-LII signals at any given moment *t* is normalized to the peak signal, yielding:

(A3)
$$\begin{array}{cc} b\left(t,\;\lambda \right) & =S\left(\lambda ,\;t\right)/S\left(\lambda ,\;t_{m}\right)\\ & =\frac{\iint p\left(N_{p}\right)p\left(d_{p}\right)N_{p}C_{\mathrm{abs},\;\lambda }\left[d_{p}\left(t\right)\right]I_{b,\;\lambda }\left[T_{p}\left(t\right)\right]d\left(d_{p}\right)d\left(N_{p}\right)}{\iint p\left(N_{p}\right)p\left(d_{p}\right)N_{p}C_{\mathrm{abs},\;\lambda }\left[d_{p}\left(t_{m}\right)\right]I_{b,\;\lambda }\left[T_{p}\left(t_{m}\right)\right]d\left(d_{p}\right)d\left(N_{p}\right)} \end{array}$$

where *t*_m_ is the time at which the spectral TiRe-LII signal *J*_λ_(*t*) reaches its maximum value, and **b**(*t*, *λ*) is the spectral normalized TiRe-LII signal at time *t*.

### Appendix A.2. Simplification of Normalized TiRe-LII Signal Simulation Using the Equivalent Thermal Accommodation Coefficient

Since the values of the shielding factor *η* and the thermal accommodation coefficient *α*_T_ are related to the structure and size of the aggregates, the combined parameter of *η* and *α*_T_, namely the equivalent thermal accommodation coefficient *α*_eff_, may be constant within an aggregate but variable across different aggregates. However, in this study, *α*_eff_ is one of the unknown parameters in the inverse problem, necessitating its constancy for all detected aggregates. In light of this, the definition of *α*_eff_ is generalized from an individual aggregate to all detected aggregates, along with the introduction of an associated equivalent aggregate size *N*_p, eff_. Consequently, the simulation of spectral TiRe-LII signals (Equation (A2)) can be transformed as follows:

(A4)
$$S\left(\lambda ,\;t\right)=\Lambda \cdot N_{p,\;\mathrm{eff}}\int p\left(d_{p}\right)C_{\mathrm{abs},\;\lambda }\left[d_{p}\left(t\right)\right]I_{b,\;\lambda }\left[T_{p}\left(t\right)\right]d\left(d_{p}\right)$$

Based on Equation A4, the simulation of normalized TiRe-LII signals (Equation (A3)) can be simplified as follows:

(A5)
$$\begin{array}{ll} b\left(t,\;\lambda \right) & =S\left(\lambda ,\;t\right)/S\left(\lambda ,\;t_{m}\right)\\ & =\frac{\Lambda \cdot N_{p,\;\mathrm{eff}}\int p\left(d_{p}\right)C_{\mathrm{abs},\;\lambda }\left[d_{p}\left(t\right)\right]I_{b,\;\lambda }\left[T_{p}\left(t\right)\right]d\left(d_{p}\right)}{\Lambda \cdot N_{p,\;\mathrm{eff}}\int p\left(d_{p}\right)C_{\mathrm{abs},\;\lambda }\left[d_{p}\left(t_{m}\right)\right]I_{b,\;\lambda }\left[T_{p}\left(t_{m}\right)\right]d\left(d_{p}\right)}\\ & =\frac{\int p\left(d_{p}\right)C_{\mathrm{abs},\;\lambda }\left[d_{p}\left(t\right)\right]I_{b,\;\lambda }\left[T_{p}\left(t\right)\right]d\left(d_{p}\right)}{\int p\left(d_{p}\right)C_{\mathrm{abs},\;\lambda }\left[d_{p}\left(t_{m}\right)\right]I_{b,\;\lambda }\left[T_{p}\left(t_{m}\right)\right]d\left(d_{p}\right)} \end{array}$$

Subsequently, by substituting the parametric expressions for the product of spectral absorption cross-section *C*_asb, *λ*_ and blackbody spectral intensity *I*_b, *λ*_ as follows:

(A6)
$$\begin{array}{ll} C_{\mathrm{abs},\;\lambda }\left[d_{p}\left(t\right)\right]\cdot I_{b,\;\lambda }\left[T_{p}\left(t\right),\;d_{p}\left(t\right)\right] & =\frac{\pi^{2}d_{p}^{3}\left(t\right)E\left(m_{\lambda }\right)}{\lambda }\cdot \frac{8\pi hc^{2}}{\lambda^{5}\cdot \left\{\exp\left[\frac{hc}{\lambda k_{B}T_{p}\left(t\right)}\right]-1\right\}}\\ & =\frac{8\pi^{3}hc^{2}d_{p}^{3}\left(t\right)E\left(m_{\lambda }\right)}{\lambda^{6}\cdot \left\{\exp\left[\frac{hc}{\lambda k_{B}T_{p}\left(t\right)}\right]-1\right\}} \end{array}$$

Equation (A5) can be further simplified as follows:

(A7)
$$\begin{array}{ll} b\left(t,\;\lambda \right) & =\frac{\int p\left(d_{p}\right)C_{\mathrm{abs},\;\lambda }\left(d_{p}\right)I_{b,\;\lambda }\left[T_{p}\left(t\right),\;d_{p}\left(t\right)\right]d\left(d_{p}\right)}{\int p\left(d_{p}\right)C_{\mathrm{abs},\;\lambda }\left(d_{p}\right)I_{b,\;\lambda }\left[T_{p}\left(t_{m}\right),\;d_{p}\left(t_{m}\right)\right]d\left(d_{p}\right)}\\ & =\int \frac{d_{p}^{3}\left(t\right)p(d_{p})}{\exp\left[hc/\lambda k_{B}T_{p}\left(t\right)\right]-1}dd_{p}/\int \frac{d_{p}^{3}\left(t_{m}\right)p(d_{p})}{\exp\left[hc/\lambda k_{B}T_{p}\left(t_{m}\right)\right]-1}dd_{p} \end{array}$$

## Appendix B

Figure A1 and Figure A2, along with Table A1, are plotted using the raw data from the 100 four-parameter inversions, where the *σ*_d_ = 1 peak has not been excluded.

**Table A1:** Four-parameter inversion results were obtained using three laser fluences when the flame temperatures were 1700 K and 1100 K, respectively.

Scenario	*F* = 0.09 J/cm^2^	*F* = 0.12 J/cm^2^	*F* = 0.15 J/cm^2^
*T*_g_ = 1700 K (Corresponding to Figure A1)			
*μ*_d_/nm	18.55 ± 0.95	18.95 ± 3.11	18.87 ± 3.53
*σ*_d_	1.26 ± 0.04	1.23 ± 0.15	1.24 ± 0.16
α_eff_	0.23 ± 0.01	0.24 ± 0.06	0.25 ± 0.06
*T*_g_/K	1603 ± 47	1626 ± 170	1635 ± 206
*T*_g_ = 1100 K (Corresponding to Figure A2)			
*μ*_d_/nm	24.69 ± 4.11	19.70 ± 2.61	20.28 ± 2.71
*σ*_d_	1.31 ± 0.16	1.21 ± 0.07	1.18 ± 0.11
α_eff_	0.31 ± 0.06	0.25 ± 0.09	0.27 ± 0.09
*T*_g_/K	1035 ± 75	982 ± 206	1062 ± 267
